# Topography-guided corneal surface laser ablation combined with simultaneous accelerated corneal collagen cross-linking for treatment of keratoconus

**DOI:** 10.1186/s12886-021-02042-x

**Published:** 2021-07-24

**Authors:** Yu Zhang, Yueguo Chen

**Affiliations:** Department of Ophthalmology, Beijing key laboratory of restoration of damaged ocular nerve, Peking University Third Hospital, 49 North Huayuan Road, Haidian District, 100191 Beijing, China

**Keywords:** Keratoconus, Topography-guided, Laser ablation, Accelerated, Corneal collagen cross-linking

## Abstract

**Background:**

to study the outcomes of topography-guided customized excimer laser subepithelial ablation combined with accelerated CXL for progressive keratoconus.

**Methods:**

Thirty-one eyes of 30 patients with progressive keratoconus were included in this prospective study. Topography-guided excimer laser ablation without refractive correction was performed. Simultaneous accelerated collagen cross-linking with ultraviolet light of 30 mW/cm^2^ for 4 min was followed. Uncorrected distance visual acuity (UCVA), manifest refraction, corrected distance visual acuity (CDVA), tomograghy were examined at postoperative 1, 6, and 12 months.

**Results:**

UDVA improved slightly after surgery (*P* > 0.05). BSCDVA improved significantly from 0.32 ± 0.20 logMAR to 0.15 ± 0.14 logMAR at postoperative 12 months (*P* < 0.05). During 12-month follow-ups, there were no significant differences in manifest refraction and corneal keratometry except for maximal keratometry value of the anterior surface (K_apex_), which decreased significantly from 57.23 ± 5.09D to 53.13 ± 4.47D (*P* < 0.05). Even though the thinnest corneal thickness decreased from 465 ± 24 μm to 414 ± 35 μm (*P* < 0.05), curvature asymmetry index front (SIf), keratoconus vertex front (KVf) and Baiocchi Calossi Versaci index front (BCVf) decreased significantly till postoperative 12 months (*P* < 0.05). Corneal higher-order aberrations and coma also decreased significantly till 12 months after surgery (*P* < 0.05).

**Conclusions:**

Topography-guided surface ablation without refractive correction combined with simultaneous accelerated collagen cross-linking provided good stability in refraction and corneal curvature, and also showed significant improvement in BSCDVA, corneal regularity and corneal optical quality.

## Background

Keratoconus is a progressive ectatic corneal disorder that results in corneal stroma impairment and biomechanical weakening. Corneal collagen cross-linking (CXL) is an effective treatment to halt the progression of keratoconus [1]. The classic Dresden CXL uses ultraviolet light of 3mW/cm^2^ illumination and a single treatment process needs 60 min to reach a total energy of 5.4 J/cm^2 ^[2]. Recently, researchers have developed accelerated CXL protocols that speed up the procedure using higher-intensity radiation. Several accelerated protocols have been reported to provide comparable results to the classic Dresden CXL [3, 4]. Furthermore, accelerated CXL has been proven to halt the progression of keratoconus in the majority of pediatric patients [5]. Despite reports of well prevention for keratoconus progression and slight improvements of keratometry following CXL, the benefits in terms of improvement in UDVA or CDVA are negligible [6, 7]. In addition, approximately 15 % of keratoconus patients cannot tolerate contact lenses and some patients have difficulty in fitting the appropriate contact lenses. Thus, some attempts have been made to not only prevent the progression of keratoconus, but also improve visual quality in keratoconus patients.

The combination of CXL and topography-guided photorefractive keratectomy (TG-PRK) was first proposed by Kanellopoulos AJ in 2007, which was known as Athens Protocol [8, 9]. Previous studies, combining different protocols of CXL and photorefractive treatment performed at the same time or in two-steps, demonstrated a significant improvement of keratometry readings and visual function [9–20]. In these previous studies, however, photorefractive ablation with partial refractive correction was used in all or part of the cases, aiming at reducing not only the lower but also the higher-order aberration and irregular corneal astigmatism [9–20]. As we know, vertical asymmetry and higher-order aberrations are the main reasons for the poor visual function of keratoconus, and lower-order aberrations can be well corrected by spectacles, soft contact lens or implantable contact lens (ICL) implantation. So, photorefractive treatment should focus on correcting only higher-order aberrations, so as to minimize the loss of corneal stroma.

In this prospective study, we examined the evolution of the visual, refractive and tomographic changes during 1-year follow-up after simultaneous TG-PRK without refractive correction followed by accelerated CXL in patients with progressive keratoconus.

## Subjects and methods

### Patients

This prospective study comprised 31 eyes of 30 patients (both eyes of 1 patient), aged between 12 and 34 years (mean, 24.3 ± 6.3 years), diagnosed as progressive keratoconus and treated by TG-PRK combined with simultaneous accelerated CXL at Peking University Third Hospital from December, 2016 to March, 2018. KC1 or KC2 was graded on the basis of Amsler and Muckenhirn standard [21]. This study received approval from the ethics committee of Peking University Third Hospital and adhered to the tenets of the Declaration of Helsinki. Written informed consent was obtained from each participant or participant’s parents before our interventions.

 The inclusion criteria were:(1)Corneal tomography and topography, clinical symptoms and signs demonstrated keratoconus, with a trend of progress in the past 12 months and met one of the following conditions: the maximum K reading increased by >1D; mean corneal refractive power increased by >1D; astigmatism increased by >1D;the spherical equivalent of manifest refraction increased by >1.0D with best spectacle corrected distance visual acuity (BSCDVA) lost more than one line; the thinnest corneal thickness decreased by >10%(2). Rigid gas permeable (RGP) contact lens intolerance or inadequate fitting(3)The thinnest thickness of cornea >450 μm and the predicted postoperative thickness of stromal bed >350 μm.

The exclusion criteria were:(1)Over mental tension or too young to well cooperate during surgery.(2)Active ocular infection or inflammation, the history of refractive surgery, herpes keratitis, and the ocular diseases other than keratoconus that seriously affected BSCDVA.(3)Prominent corneal scarring and BSCDVA >1.30 logMAR.(4)Definitively diagnosed and uncontrolled auto-immune diseases or connective tissue diseases.(5)The women in pregnant or lactating period.

### Preoperative Examinations

All patients had a full ophthalmological examination, including uncorrected distance visual acuity (UDVA), cycloplegic and manifest refractions, BSCDVA, slit-lamp evaluation, Goldmann applanation tonometry, and fundoscopy examinations. With the Sirius combined topographer and tomographer (CSO, Italy), the following parameters were evaluated: maximal keratometry value of the anterior surface (K_apex_), flat-axis keratometric value (K1), steep-axis keratometric value (K2), corneal astigmatism, minimum corneal thickness (ThkMin), curvature symmetry index front (SIf), keratoconus vertex front (KVf), Baiocchi Calossi Versaci index front (BCVf), root-mean-square of the total higher-order aberrations (HOA-RMS), coma (Coma-RMS), and spherical aberration (SA-RMS). Corneal topography data for topography-guided customized ablation were obtained from the placido-based topographer (Vario Topolyzer, Alcon, USA). Corneal endothelial cell density (ECD), hexagon cell percentage (HEX) and cell area variation coefficient (VC) were examined by specular microscopy (Topcon, Japan).

### Surgical procedures

**Figure 1:**
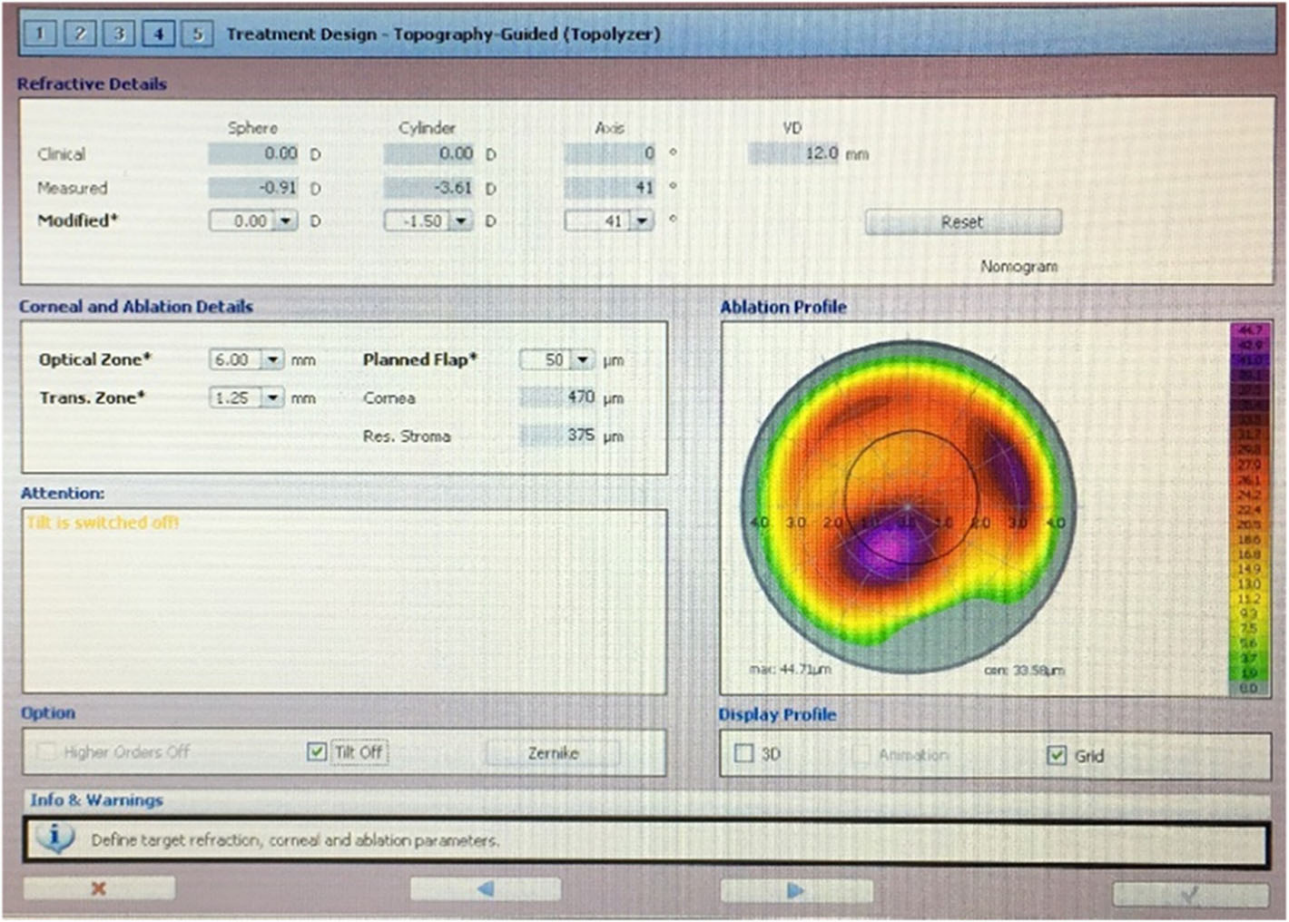
.

All treatments were performed by the same surgeon (YG Chen). Under topical anesthesia, fresh prepared 20 % alcohol was instilled into the epithelial trephine with a diameter of 9.0 mm, soaking for 20 s. Then the corneal epithelium was peeled and removed. TG-PRK was performed using the Topography-Guided (Topolyzer) software of WaveLight EX500 excimer laser system (Alcon, USA) with an optic zone of 5.0 ~ 6.0 mm. TG-PRK referred to the correction of neither refractive sphere nor cylinder, but 0 ~ -2.25D of compensated (“measured”) cylinder which was automatically calculated based on the Topolyzer topographic results. The ablation depth was calculated automatically by EX500, and controlled within 50 μm by reducing the optical zone to some extent. (Fig. 1) Mean maximum ablation depth was 42.16 ± 2.11 μm (28 ~ 50 μm). Before and during laser ablation, the static cyclotorsion and kappa angle of the eye were automatically compensated on the basis of the topographic examination. The ablation center was automatically set to the corneal vertex. Mitomycin C was not used. Irrigation with balanced salt solution was followed by corneal soaking with riboflavin (0.1 % riboflavin sodium phosphate ophthalmic solution VibeX Rapid; Avedro, Inc) for 10 min. The cornea was irradiated for 4 min by ultraviolet light (30mW/cm^2^, KXL ultraviolet instrument, Avedro, USA) with a total energy delivered of 7.2 J/cm^2^. A bandage soft contact lens was applied until complete epithelialization.

### Postoperative treatment and follow up

Levofloxacin 0.5 % eye drops (Santian Pharmaceutical co., LTD, Japan) were applied 4 times a day until epithelial healing. Fluorometholone 0.1 % eye drops (Santian Pharmaceutical co., LTD, Japan) were applied 4 times a day in the first post-operative week and tapered weekly for 4 weeks. After contact lens removal, artificial tears or lubricants were used for at least 1 month according to the conditions of dry eye or delayed healing of epithelium.

The follow-up time points were 1 day, 5 days, 14 days, 1 month, 3 months, 6 months and 12 months after surgery. UDVA and slit lamp examination were performed at every time point. Corneal haze was evaluated according to the system reported by Fantes et al.[22] At 1-month, 6-month and 12-month follow-up, manifest refraction, BSCDVA, Sirius combined topographer and tomographer, Topolyzer topographer were examined. At 1-month follow-up, specular microscopy and anterior segment optical coherence tomography (AS-OCT) (Visante OCT; Carl Zeiss Meditec) were examined. Demarcation line depth was measured at corneal vertex using AS-OCT by an experienced technician.

### Statistical analysis

SPSS 21.0 statistical software (SPSS, Inc., Chicago, IL) was used to analyze the data. Continuous data were expressed as mean values ± standard deviation. Visual acuity was converted to logMAR for statistical analysis. Repeated measurement variance analysis was used for the comparison of the overall difference of continuous parameters among preoperative and postoperative multiple time points. Dunnett-t test was used for comparing parameters between different two time points. Pearson Chi-Square test was used to compare BSCDVA change between different time points. *P*<0.05 was considered to be statistical significant.

## Results

### Visual acuity

UDVA and BSCDVA detailed data are illustrated in Table 1. There was no significant difference in UDVA between preoperative and postoperative time points (*P* > 0.05). BSCDVA improved significantly from baseline to postoperative 1-month and 6-month (*P* < 0.05), and remained relatively stable till postoperative 12 months (*P* > 0.05).

**Figure 2:**
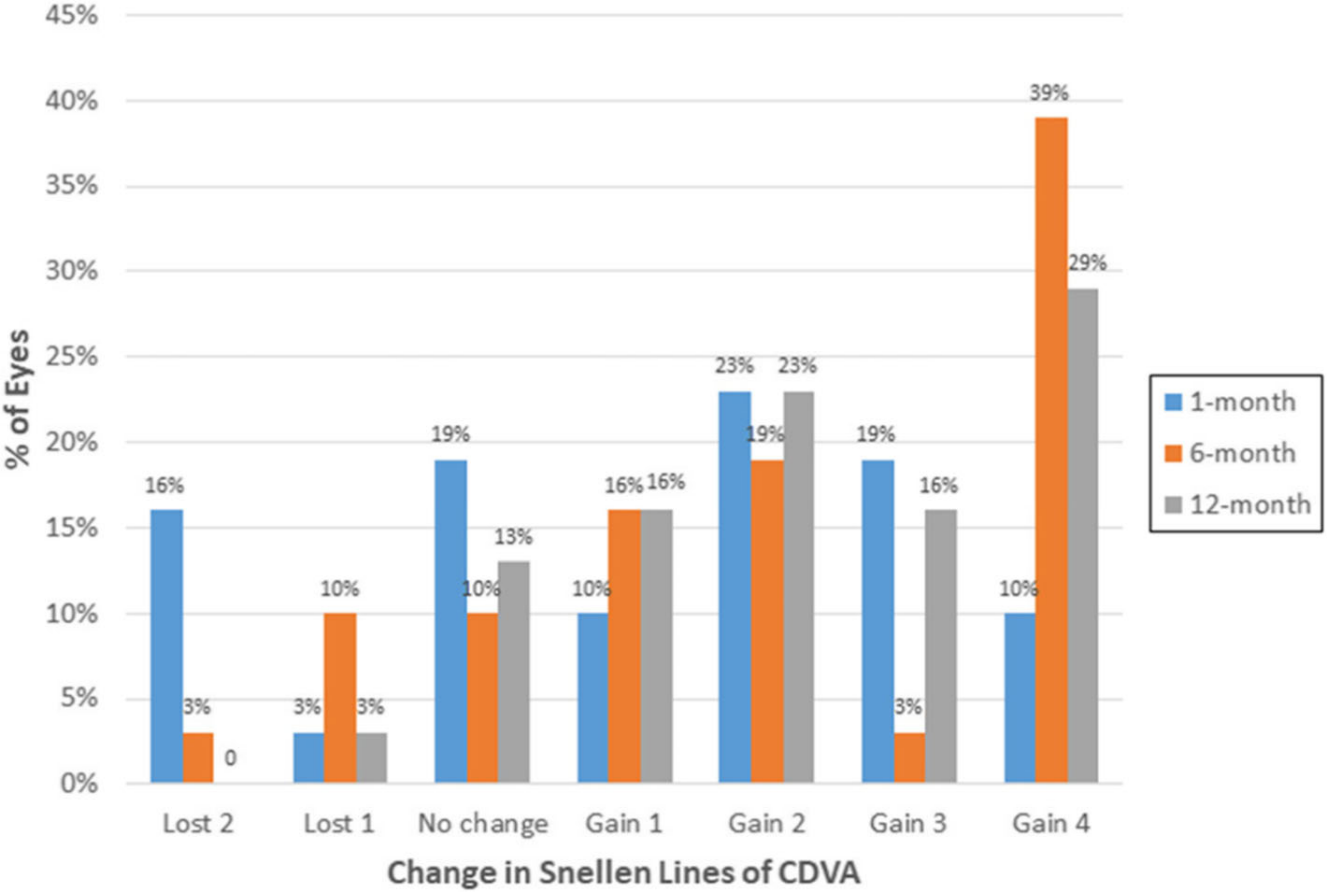
.

At postoperative 1-month, BSCDVA lost 2 lines in 5 eyes (16 %), 1 line in 1 eye (3 %), unchanged in 6 eyes (19 %) and increased over 2 lines in 16 eyes (52%). As time went by, BSCDVA improved gradually. At postoperative 12-month, only 1 eye lost 1 line of BSCDVA, and BSCDVA unchanged in 4 eyes (13 %), and increased over 2 lines in 21 eyes (68 %) (*P* < 0.05).(Fig. 2)

**Table 1:** Mean changes in visual acuity over 12- month follow-up (n = 31, x ± s)

	Preoperative		1-month	6-month	12-month	F	*P*
UDVA(LogMAR)	0.79 ± 0.33		0.76 ± 0.23	0.70 ± 0.27	0.71 ± 0.27	2.016	*0.134*
BSCDVA(LogMAR)	0.32 ± 0.20		0.24 ± 0.24*	0.16 ± 0.22*^#^	0.15 ± 0.14*^#^	13.495	*< 0.001*

Abbreviations: *UDVA* uncorrected distance visual acuity; *BSCDVA* best spectacle corrected distance visual acuity

* significant difference compared with preoperative baseline (*P* < 0.05)

^**#**^ significant difference compared with postoperative 1-month (*P* < 0.05)

### Manifest Refraction

Table 2 shows the manifest refraction values during follow-ups. There were no significant overall differences in sphere, cylinder and spherical equivalent during the 12-month follow-up (*P* > 0.05). Only cylinder at postoperative 6 and 12 months decreased significantly, compared to the preoperative value (*P* < 0.05).

**Table 2:** Mean changes in manifest refraction over 12-month follow-up. (*n* = 31, x ± s)

	Preoperative	1-month	6-month	12-month	F	*P*
Sphere(D) Cylinder(D) SE(D)	-5.56 ± 4.14 -3.75 ± 1.53 -7.43 ± 4.52	-5.16 ± 4.09 -3.43 ± 2.12 -6.87 ± 4.29	-5.30 ± 4.44 -3.21 ± 1.85* -6.92 ± 4.78	-5.19 ± 4.35 -3.07 ± 1.96* -6.65 ± 4.67	0.478 1.810 1.115	0.700 0.168 0.360

Abbreviations: *SE* spherical equivalent

* significant difference compared with preoperative baseline (*P* < 0.05)

### Corneal curvature

**Figure 3:**
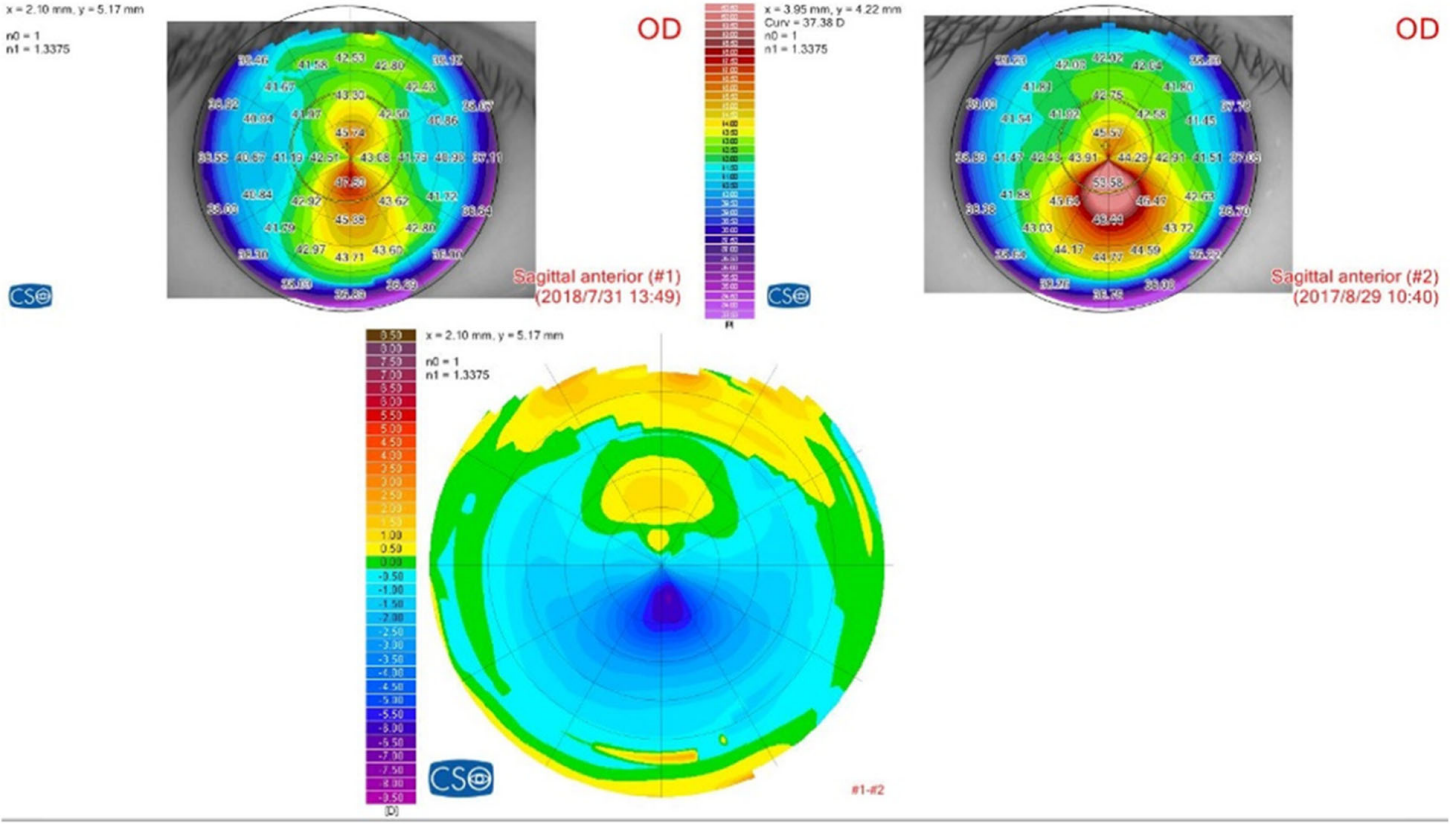
.

Table 3 shows corneal curvature parameters detected by Sirius combined topographer and tomographer during follow-ups. K_apex_ decreased significantly at all visits compared to the previous value and the preoperative value (*P* < 0.05). At 12-month visit, mean K_apex_ decreased to 53.13 ± 4.47D, compared to the preoperative mean K_apex_ of 57.23 ± 5.09D (*P* < 0.05). Although the overall differences in K2 and mean K during follow-up were significant, only values at 6 and 12 months were significantly different from 1-month values (*P* < 0.05). There was no significant difference in corneal cylinder during 12-month follow-ups (*P* > 0.05). The corneal topography change of a typical case is shown in Fig. 3.

**Table 3:** Mean changes in corneal curvature parameters over 12-month follow-up (*n* = 31, x ± s)

	Preoperative	1-month		6-month	12-month		F	*P*
K_apex_(D) K1(D) K2(D) mean K(D) CYL(D)	57.23 ± 5.09 45.47 ± 2.35 48.35 ± 2.94 46.86 ± 2.53 -2.87 ± 1.54	54.96 ± 4.44* 45.64 ± 2.71 48.73 ± 3.47 47.12 ± 2.97 -3.09 ± 1.74		54.24 ± 4.87* 45.31 ± 2.83^**#**^ 48.23 ± 3.26^**#**^ 46.69 ± 2.98^**#**^ -2.91 ± 1.61	53.13 ± 4.47*^#&^ 45.28 ± 2.77^**#**^ 48.09 ± 3.04^**#**^ 46.63 ± 2.79^**#**^ -2.79 ± 1.60		29.453 1.794 4.077 3.775 1.707	< 0.001 0.171 0.016 0.022 0.188

Abbreviations: *K*_apex_ maximal keratometry value of the front surface; *K* flat-axis keratometric value; *K2* steep-axis keratometric value; *Mean K* mean keratometric value; *CYL *corneal cylinder

* significant difference compared with preoperative baseline (*P* < 0.05)

^**#**^ significant difference compared with postoperative 1-month (*P* < 0.05)

^&^ significant difference compared with postoperative 6-month (*P* < 0.05)

### Keratoconus parameters

Table 4 shows keratoconus parameters detected by Sirius combined topographer and tomographer during follow-ups. SIf decreased significantly at postoperative 1 month and remained stable until postoperative 12 months (*P* < 0.001). KVf began to decrease significantly at 6 months after operation and continued to decrease till 12 months after operation (*P* < 0.001). BCVf decreased significantly at postoperative 6 months and remained stable until postoperative 12 months (*P* < 0.001). ThkMin decreased significantly at postoperative 1 month, and increased continuously from postoperative 6 months to 12 months (*P* < 0.001).

**Table 4:** Mean changes in keratoconus parameters over 12-month follow-up (*n* = 31, x ± s)

	Preoperative	1-month	6-month	12-month	F		*P*
SIf (D)	5.65 ± 3.12	3.64 ± 3.20*	3.62 ± 2.66*	3.30 ± 2.82*	21.950		< 0.001
KVf (µm)	30.32±12.62	31.00±13.89	27.97±12.66*^#^	26.77±12.23*^#&^	19.741		< 0.001
BCVf (μm)	2.93±1.29	2.88±1.48	2.47±1.48*^#^	2.49±1.25*^#^	8.357		< 0.001
ThkMin (μm)	465 ± 24	387 ± 34*	400 ± 32*^#^	414 ± 35*^#&^	75.081		< 0.001

Abbreviations: *SIf* curvature symmetry index front; *KVf* keratoconus vertex front; *BCV* Baiocchi Calossi Versaci index front; *ThkMin* thinnest corneal thickness

* significant difference compared with preoperative baseline (*P* < 0.05)

^**#**^ significant difference compared with postoperative 1-month (*P* < 0.05)

^&^ significant difference compared with postoperative 6-month (*P* < 0.05)

### Corneal aberration

Corneal HOA and coma decreased significantly at postoperative 1 month, and decreased continuously until 12 months after operation (*P* < 0.001). However, spherical aberration increased significantly at postoperative 1 month, and returned to the preoperative level at postoperative 6 and 12 months (*P* < 0.05). (Table 5)

**Table 5:** Mean changes in corneal aberration parameters over 12-month follow-up (*n* = 31, x ± s)

	Preoperative	1-month	6-month	12-month		F	*P*
HOA-RMS	2.25 ± 0.84	2.02 ± 0.98*	1.71 ± 0.85*^#^	1.57 ± 0.85*^#&^		32.796	< 0.001
Coma-RMS	1.82±0.78	1.46±0.93*	1.26±0.81*^#^	1.14±0.79*^#&^		21.115	< 0.001
SA-RMS	0.45 ± 0.36	0.78 ± 0.55*	0.54 ± 0.40^**#**^	0.54 ± 0.39^**#**^		5.982	0.003

Abbreviations: *HOA-RMS* root-mean-square of the total higher-order aberrations; *Coma-RMS* root-mean-square of coma; *SA-RMS* root-mean-square of spherical aberration

*: significant difference compared with preoperative baseline (*P* < 0.05)

^**#**^: significant difference compared with postoperative 1-month (*P* < 0.05)

^&^: significant difference compared with postoperative 6-month (*P* < 0.05)

### 3.6 Corneal endothelial cell

There were no significant differences in ECD, HEX and cell area VC at postoperative 1 month, compared to the preoperative values (*P* > 0.05). (Table 6)

**Table 6:** Mean changes in corneal endothelial cell parameters at 1-month follow-up (*n* = 31, x ± s)

	Preoperative	1-month	t	*P*
ECD HEX(%) VC (%)	2953.24 ± 120.38 61.64 ± 5.82 33.41 ± 1.60	2994.81 ± 72.51 54.00 ± 3.96 33.49 ± 1.94	-0.493 1.279 -0.065	0.633 0.230 0.949

Abbreviations:* ECD* endothelial cell density; *HEX* hexagon cell percentage; *VC* cell area variation coefficient

### Postoperative corneal changes and complications

At postoperative 1 month, the demarcation line was well defined in each case with a mean depth of 216 ± 25*µ*m. Aseptic inflammatory sub-epithelial infiltration occurred in one eye (3.2 %). No extra specific treatment was used and the opacity was basically absorbed within 4 weeks. At postoperative 1 month, corneal haze started to appear and dissipated gradually from postoperative 3 months to 12 months. The number of eyes with haze of grade 0, 0.5, 1, and 2 at postoperative 12-month follow-up were 25 eyes (80.6 %), 4 eyes (13.0 %), 1 eye (3.2 %), and 1 eye (3.2 %), respectively.

## Discussion

The classic Dresden CXL using ultraviolet light of 3mw/cm^2^ illumination is a time-consuming procedure, which can cause high percentage of haze [19] and the risk of progressive corneal flattening [20], especially in simultaneous combined treatments. The accelerated CXL uses ultraviolet light of high irradiation intensity. According to the Bunsen-Roscoe law of photochemical effect, the higher the illumination, the shorter the exposure time [23].The exposure time required is greatly shortened, which improves the treatment efficiency and increases patients’ compliance. Additionally, accelerated CXL was proved to be effective and have fewer complications [4, 5]. So, recently, accelerated CXL has been widely used by most surgeons for CXL combined treatment [13, 16–18, 20]. In the current study, 30mW/cm^2^ ultraviolet light intensity of illumination for 4 min was used for the first time.

Initially, epithelial removal prior to CXL was performed using manual debridement (with or without alcohol) [13–15]. Recently, transepithelial phototherapeutic keratectomy (PTK) has also been employed as a method for removing the epithelial [16–18]. Because of a doughnut-shaped model of the corneal epithelium in keratoconus [24], PTK removes some stromal tissue from the central cone, which flattens the cornea more than manual debridement [25], but consumes more corneal stromal tissue at the cone apex. The corneal surface following PTK is more consistent with the preoperative topography, which makes the subsequent TG-PRK become more accurate. PTK epithelial removal leaves some epithelium around the cone, which may reduce the ablation volume of the corneal stromal tissue during TG-PRK, but can cause under-correction. In the present study, manual debridement with alcohol was used, which could reduce the loss of corneal stroma at the cone apex to the greatest extent, and reduced the excessive friction stimulation of corneal stroma by mechanical scraping.

Topography-guided customized ablation attempts to maintain the aspheric shape of the cornea and neutralize corneal irregularities [26]. It has been shown to be effective in treating irregular astigmatism caused by iatrogenic corneal irregularities[27]. Since topography-guided ablation for normalizing the anterior cornea can bring in refractive change, especially astigmatism change [28], the clinical refraction should be adjusted to keep it neutral after refractive correction. Kanellopoulos found that topography-modified refraction (TMR) offered superior refractive and visual outcomes to standard clinical refraction in myopic topography-guided LASIK [29]. In the current study, we used TMR for reference to compensate for partial cylinder measured by topographer without refractive correction while topography-guided ablation of irregular corneas on the basis of ensuring the depth of corneal ablation.

In the current study, UDVA improved slightly after surgery, but there was no statistical significance. BSCDVA improved significantly from 0.32 ± 0.20 logMAR to 0.15 ± 0.14 logMAR at postoperative 12 months (*P* < 0.05). Manifest refraction, flat K, steep K and corneal cylinder all decreased slightly after surgery, but no statistical significance was found. However, K_apex_flattened significantly after surgery. Generally speaking, the improvement of UDVA, the decrease of refraction and corneal curvature were not obvious, which were different from most previous studies [10–20]. Because of partial correction of clinical refraction, there were improvements in UDVA [8–13, 15–18], curvature readings [13, 15, 17–20] and manifest refraction [13, 15, 16, 18–20] after surgery. The CXL plus TG-PRK in the present study aimed at halting the progression of keratoconus and reducing corneal HOA, so we did not carry out refractive correction, which was a main difference between the present study and previous studies [10–20]. Despite of the different TG-PRK protocol, the present study still showed improvements in BSCDVA and K_apex_similar to the previous studies [10–20], which increased the correction effect of frame glasses or ICL implantation when contact lenses were not tolerated or helped to regain a good contact lenses fitting.

The current study also showed that SIf, KVf and BCVf, which reflected the irregularity of the anterior corneal surface, reduced significantly after surgery and remained in a reduced state up to 12 months after surgery. Those indicated that the cornea became regular after surgery and resulted in the improvement of BSCDVA. Some previous studies showed similar results that index of surface variance (ISV) and index of height decentration (IHD) decreased significantly after surgery[17]. The current study also found that corneal aberrations including corneal HOA and coma decreased continuously within 12 months after surgery, which also indicated the improvement of corneal regularity due to TG ablation. Ahmet et al.[16] and Rechichi et al. [18] found that corneal HOA, coma and spherical aberration (SA) all significantly decreased at 24 months after surgery. The difference of the change of corneal SA might related to the different excimer laser system (Schwind Amaris, Germany) and different ablation mode (Transepithelial TG-PRK).

Like some previous studies [10, 12, 16, 18], MMC was not used in the present study. Kymionis GD et al. found that CXL could destroy the regeneration of corneal anterior stromal cells by confocal microscopy, so it was not necessary to use MMC after PRK combined CXL [30]. The synergistic effect of CXL and MMC may cause more cell death and more corneal haze [19].Although haze was observed at each case in postoperative one month in the present study, it gradually faded away 3 to 12 months after operation. This slightly obvious haze may be related to alcohol deepithelialization and 4-week treatment of glucocorticoid eyedrops after surgery.

The limitations of this study are as follows. First, this study had a small sample and was lack of a control group. Second, we didn’t estimate the impact of corneal posterior surface when planning TG ablation. In future, software based on calculation of mean pupillary power or raytracing technology compensating anterior and posterior corneal surfaces refractive and aberrometric contributions are mandatory to optimize visual and refractive outcomes. Third, like most previous studies, we used uniform protocol in all cases. Pachymetry-based or topography-guided customized CXL might be more effective and safer [18, 31, 32]. Rechichi et al.[18]offset ablation center towards the location of cone apex, which might better reduce coma. These customized procedures provided a good reference for our surgical protocol in the future.

It has been reported that a small number of keratoconus (around 8 %) may still progress after CXL [33]. Thus regular and long-term follow-up of refraction and corneal topography/tomography at different time points after surgery is necessary, especially after the combined surgery with reduced corneal thickness. The research of long-term safety is warrant.

## Conclusions

In conclusion, the current study showed the significant improvement in BSCDVA, K_apex_, corneal irregularity indices and RMS of HOA and coma, and also showed good stability in refraction and corneal curvature after simultaneous TG-PRK without refractive correction followed by accelerated CXL (30mW/cm^2^) in patients with progressive mild-to-moderate keratoconus. The treatment protocol is convenient, time-saving, effective and safe. However, large-scale, comparative, long-term trials are required to determine the optimum parameters and evaluate the long-term safety and effectiveness of this combined surgery.

## Data Availability

The datasets generated and/or analysed during the current study are not publicly available due to limitations of ethical approval involving the patient data and anonymity but are available from the corresponding author on reasonable request.

## Abbreviations

CXL: Corneal collagen cross-linking
UCVA: Uncorrected distance visual acuity
CDVA: Corrected distance visual acuity
SIf: Curvature symmetry index front
KVf: Keratoconus vertex front
BCVf: Baiocchi Calossi Versaci index front
TG-PRK: Topography-guided photorefractive keratectomy
PTK: Phototherapeutic keratectomy
BSCDVA: Best spectacle corrected distance visual acuity
K_apex_: Maximal keratometry value of the anterior surface
K1: Flat-axis keratometric value
K2: Steep-axis keratometric value
ThkMin: Minimum corneal thickness
HOA-RMS: Root-mean-square of the total higher-order aberrations
Coma-RMS: Root-mean-square of coma
SA-RMS: Root-mean-square of spherical aberration
ECD: Corneal endothelial cell density
HEX: Hexagon cell percentage
VC: variation coefficient
AS-OCT: Anterior segment optical coherence tomography
ICL: Implantable contact lens
